# What Should the Medicine-case Hold?

**Published:** 1904-11

**Authors:** 


### WHAT SHOULD THE MEDICINE-CASE HOLD?

We have recently published in our correspondence columns an article in which a general practitioner, practicing in a country district, saw fit to enumerate the remedies which he had found most useful in the medicine-case which he found necessary to carry on his daily rounds. The physician who lives in a city or large town usually depends entirely upon prescriptions for the dispensing of his remedies, although at the present time, in many instances, it is found convenient, even by the city practitioner, to carry a small case in which certain common remedies may be found. It is manifest, on the one hand, that the country practitioner must carry with him a sufficiently large assortment of drugs to meet all the indications which will come to him in a diversified practice. Yet, on the other hand, it is certain that he who carries an unnecessarily large number of remedies will soon find that he is overburdening himself, and after several months perceive that some of his bottles have remained untouched. Probably no physician of experience has failed to be surprised at finding that certain drugs which he placed in his case have scarcely been used, while others have had to be renewed several times within a week. It is evident, therefore, that a comparatively small number of remedies are in constant demand, and it is with these that we propose to deal, although doubtless certain physicians will feel that some of the remedies which are not named deserve a place in this list.

Of the emergency medicines suitable for a medicine-case may be named hypodermic tablets of cocaine hydrochlorate, which can be used in extracting foreign bodies from the eye and for other local anesthetic purposes, including minor operations. These tablets will usually be of from one-eighth to one-quarter of a grain in strength. The second line of tablets are uncompressed tablet triturates of bichloride of mercury for the preparation of antiseptic solutions. Thirdly, we should mention acetanelid in powder, in which form it may be used for the relief of neuralgic pain, for the arrest of obstinate vomiting, as a surgical dressing, and as an antipyretic. Fourthly, there may be named hypodermic tablets of one-quarter of a grain of morphine sulphate, which can either be given by the mouth or employed hypodermically for all the various purposes for which we employ this important drug. Lastly, hypodermic tablets of one-twentieth of a grain of strychnin should also be included in this list for obvious reasons. Of the remedies which can be used for subacute or ordinary conditions, calomel of course stands at the head of the list, and it is probably wise to have it in plain tablets and also in tablets combined with ipecac. Not infrequently calomel fails to produce the prompt effects which we desire because this valuable stimulant to the intestinal glands is not combined with it. Yet, on the other hand, there are some cases in which a dose of calomel must be so large that were it combined with ipecac too great an ipecac influence would be produced. In the same class of remedies should be included Dover's powders in two or three-grain tablets, and tablets of the fluid extract of aconite in the dose of one-tenth of a minim, since by this means a quantity suitable for a child can readily be dispensed, and by giving several tablets a full aconite influence can be produced in an adult. Still another tablet, or pill, which must find a place in every case is the well-known formula containing aloin, strychnin, and belladonna, and in addition it may be well to have another tablet or pill containing the ingredients of the compound cathartic pill of the United States Pharmacopœia, or the so-called "vegetable cathartic pill" made under the same authority. Another tablet which is exceedingly useful is that which is known as the chlorodyne tablet, which can be used for the relief of pain and the control of sharp attacks of diarrhea. Under the name of "Rhinitis Tablets" there is now marketed by almost all manufacturers a preparation composed of a small quantity of quinine, camphor, and belladonna which is useful in the early stages of an acute cold, and which may often be employed in association with Dover's powder in tablet form for breaking an acute cold in its early stages. It is hardly necessary to state that quinine, in soft capsules, or easily dissolved pills, and not in compressed tablets, must find a place in this collection.

There still remain two other tablets which will prove useful in a large number of cases, namely, those which contain digitalis alone, and those which are composed of the well-known formula consisting in digitalis, nitroglycerin, strophanthus, and belladonna. Last, but not least, every case should contain a tablet, or pill, of nitroglycerin. The advantage of having a hypodermic tablet of this substance is not only that it can be given hypodermically, but also that by dropping it in water and using a teaspoonful of the solution every hour an effect can be exercised upon the circulation which is practically identical with that which we produce in children by the employment of sweet spirits of nitre. For the physician who practices obstetrics, and what general practitioner does not, ergot, in tablet or liquid form, preferably in the form of ergone, will of course be necessary. Of preparations which will be found useful in more chronic conditions several stand preeminent. The first of these may be said to be the protiodide of mercury in one-quarter grain tablets; the second iodide of potassium or strontium; the third, bromide of potassium.

In conclusion, we may say a word in favor of the ordinary tablet triturate as against the compressed tablet triturate, which, in the great majority of instances, has to be compressed to such a degree of hardness that it is slowly dissolved in the normal stomach, and often not dissolved at all in the digestive tract of the patient who is ill. A very large number of other remedies may be added to this list, but these are the ones which the young physician must provide himself, and to these may be added others as experience teaches him his additional needs.—*Therapeutic Gazette.*

